# Boosting lignan-precursor synthesis in yeast cell factories through co-factor supply optimization

**DOI:** 10.3389/fbioe.2022.1079801

**Published:** 2022-12-02

**Authors:** Jennifer Perrin, Sébastien Besseau, Nicolas Papon, Vincent Courdavault

**Affiliations:** ^1^ Biomolécules et Biotechnologies Végétales, Université de Tours, Tours, France; ^2^ University Angers, University Brest, IRF, SFR ICAT, Angers, France

**Keywords:** cofactor, yeast cell factories, metabolic engineering, lignan, NADPH

## Introduction

Plants constitute one of the largest sources of natural products (NPs) whose biological activities have been exploited by humans for agronomic, cosmetic or pharmacological purposes. For instance, many of these NPs became essential components of the therapeutic arsenal deployed against cancers since many years, such as the lignan-derived podophyllotoxin produced by the mayapple *Podophyllum hexandrum* (Buss et al., 1995). However, NPs are usually accumulated in minute amounts in plants and their complex structures hinder total chemical synthesis at least at an industrial scale. While extraction and purification procedures have been improved and combined to semi-synthetic approaches, many plant drugs still exhibit high production costs and a supply mostly based on the exploitation of the natural resources. This engenders a huge pressure on the NP producing plants, many of which suffer from overexploitation, causing a recurring shortage of drugs currently used in the clinic. In order to secure NP supply, new strategies of production have emerged such as those relying on the transfer of genes in heterologous organisms and the metabolic engineering (Carqueijeiro et al., 2020).

**FIGURE 1:**
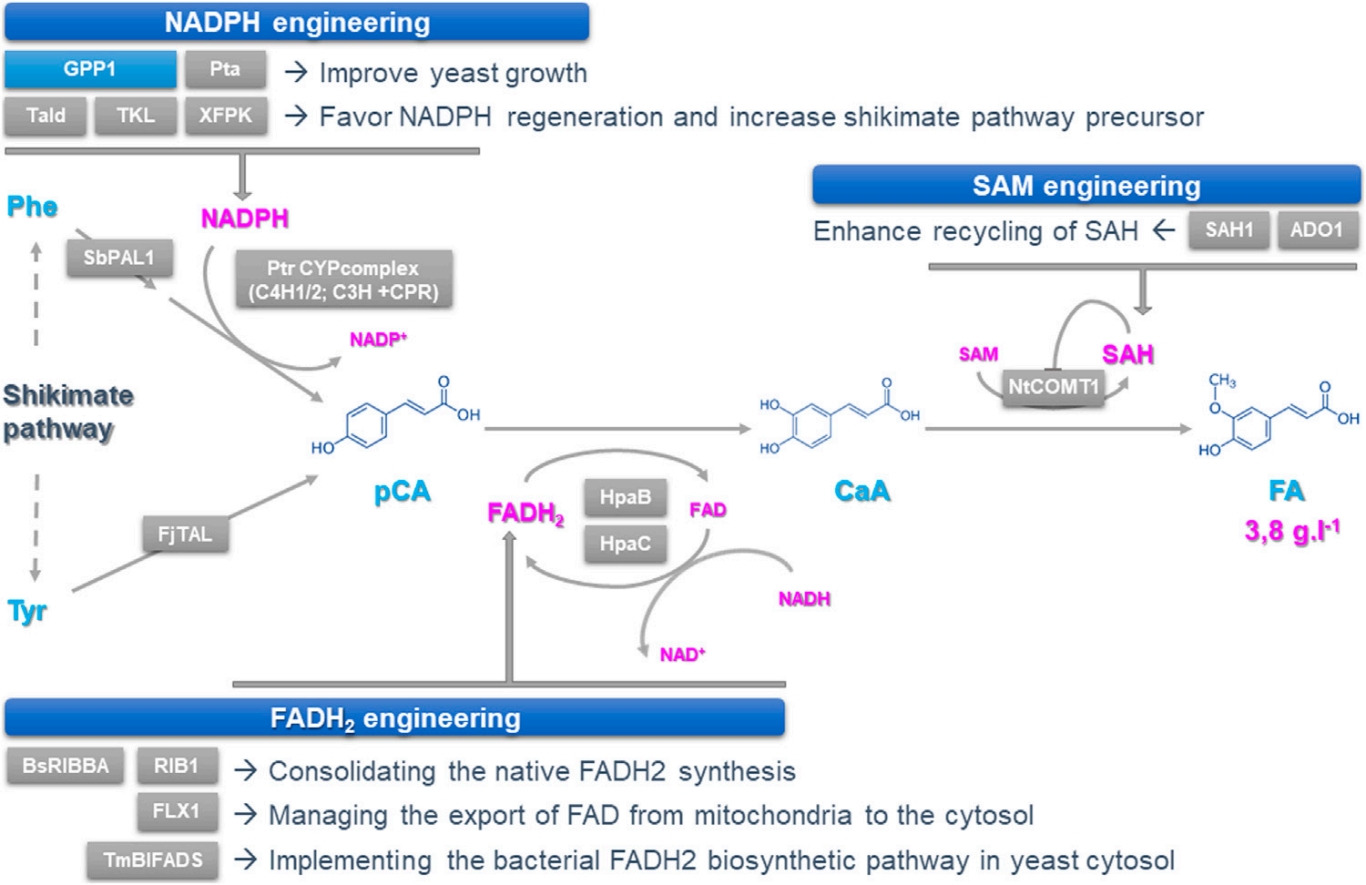
Improving the synthesis and regeneration of cofactors to increase the synthesis of podophyllotoxin precursors in yeast. Podophyllotoxin biosynthesis can be achieved through a three-step route involving deamination of tyrosine and phenylalanine to form p-coumaric acid (pCA), which is then hydroxylated and *O*-methylated to from caffeic acid (CaA) and ferulic acid (FA), respectively. Each step catalyzed by heterologous enzymes requires cofactors including NADPH, FAD (H_2_) and SAM whose supplies have been engineered by optimizing their synthesis and/or recycling. Overexpressed and deleted genes are indicated with grey and blue boxes respectively. ADO1: Adenosine kinase; BsRIBBA: *Bacillus subtilis* bifunctional DHBP synthase/GTP cyclohydrolase II; CaA: Caffeic Acid; CPR: Cytochrome P450 Reductase; FA: Ferulic Acid; FAD (H2): Flavine Adenine Dinucleotide; FjTAL: *Flavobacterium* johnsoniae tyrosine ammonia lyase; FLX1: mitochondrial FAD exporter; GPP1: GAP Phosphatase; HpaB: FAD-dependent 4-hydroxyphenylacetate-3-monooxygenase; HpaC: NADH-flavin oxidoreductase; NADP(H): Nicotinamide Adenine Dinucleotide Phosphate; NtCOMT1: *Nicotiana tabacum* CaA O-methyltransferase 1; pCA: p-Coumaric Acid; Phe: Phenylalanine; Pta: phosphotransacetylase; PtrC3H: *Populus trichocarpa* coumarate-3-hydroxylase; PtrC4H1/2: *P. trichocarpa* cinnamic acid hydroxylase ½; RIB1: GTP cyclohydrolase II; SAH: S-adenosy-l-homocysteine; SAH1: SAH hydrolase; SAM: S-adenosyl-l-methionin; SbPAL1: *Isatis indigotica* phenylalanine ammonia lyase 1; Tald: transaldolase; TKL: transketolase; TmBIFADS: *Thermotoga maritima* bifunctional riboflavin kinase/FAD synthase; Tyr: Tyrosine; XFPK: phosphoketolase.

Based on the pioneering production of the semi-synthetic production of the antimalarial artemisinin (Paddon and Keasling, 2014), metabolic engineering approaches relying on the creation of microbial cell factories have been continuously developed over the last 10 years. Through the transfer of plant genes into diverse heterologous organisms such as yeast, successful productions of valuables NPs have been achieved including resveratrol, a grape wine polyphenol displaying antioxidant properties (Li et al., 2015), ginsenosides Rh2 and R3 with promising properties against cancers (Wang et al., 2015), cannabinoids Δ9-tetrahydrocannabinol (THC) and cannabidiol (CBD) from hemp known for their beneficial psychoactive effects on human health (Gülck et al., 2020), and monoterpene indole alkaloids (MIAs) from the Madagascar periwinkle whose prominent members vincristine and vinblastine exhibit anticancer activities (Brown et al., 2015). While most of these NPs results from *de novo* synthesis, bioconversion approaches, relying on the feeding of the cell factories with an easily accessible precursor, has been also explored with success to produce vindoline used for vincristine synthesis (Kulagina et al., 2021a). One of the main challenges of metabolic engineering is obtaining high production titers compatible with industrial productions. While a production of tetrahydroisoquinoline alkaloids reaching up to 4.6 g ^−1^ has been reported in yeast (Pyne et al., 2020), many NP syntheses by engineered yeast still require dramatic increases in production yields to replace or supplement plant-based extractions. Many of the previously reported improvements involves modifications of the molecular scaffold of the yeast as well as the transferred plant genes (Guirimand et al., 2021). In an elegant study (Chen et al., 2022), the Zhou’s group recently describes a distinct approach relying on the efficient recycling of enzyme co-factors in yeast to fulfill the synthesis of caffeic acid (CaA) and ferulic acid (FA), two precursors of podophyllotoxin (Figure 1). Through this commentary article, we highlight the outstanding work of this research group and discuss how the cofactor recycling approach will undoubtedly pave the way for future high titer production of valuable NPs in yeast.

## State of the art of strategies of natural product bioproduction improvement

While the initial production of hydrocortisone in yeast reached the outstanding yield of 25 g L^−1^ (Szczebara et al., 2003), most of the plant NPs resulting from complex biosynthetic routes were only produced at a µg or mg per liter titer, thus highlighting the need of improving biosynthesis in the engineered yeasts. In recent years, several works highlighted new strategies for improving these bioproduction yields. Obviously, controlling gene expression level is one of the evident levers to operate by selecting the best yeast genomic environment such as hot-spot (Mikkelsen et al., 2012), by using strong promoters and adapted terminators (Wang et al., 2019; Liu R. et al., 2020) or by improving the translation efficiency of the heterologous genes through expression of yeast-optimized codon sequences (Lanza et al., 2014). Handling the copy ratio of the transferred gene also constitutes a powerful option as recently reported for vindoline production. By increasing the copy number of specific genes along the pathway, production bottlenecks have been alleviated as well as the synthesis of by-product resulting from enzyme promiscuity (Kulagina et al., 2021a). In addition, the identification of more efficient enzyme isoforms or engineered variant has also provided great improvements as reported for benzylisoquinoline alkaloids (Payne et al., 2021). In parallel, adapting yeast primary metabolism is also a major prerequisite as reported with the expression of truncated HMGCoA reductase gene, which encodes a non–feedback regulated rate-limiting enzyme of the mevalonate pathway for terpenoid production (Ro et al., 2006; Brown et al., 2015) or by knocking out genes encoding yeast enzymes from the Ehrlich pathway which divert tetrahydroisoquinoline alkaloid precursor from the synthesis of the final expected compound (Payne et al., 2021). Finally, spatial reconfiguration/adaptation of parts of biosynthetic pathways can be achieved as reported for transporters of tropane alkaloids (Srinivasan and Smolke, 2020) or the transfer of the whole mevalonate pathway in peroxisome for monoterpene production. Indeed, sequestration of enzymes in the peroxisomes can also increase the proximity between enzymes and their substrates in this specific cell compartment (Liu G.S. et al., 2020; Kulagina et al., 2021b) or limit cytotoxicity that some overexpressed enzyme may display (Grewal et al., 2021). Based on a similar reasoning, improving extracellular transports can limit the poisonous effects of heterologous produced metabolites and thus act as a detoxification mechanism. This is of importance when metabolites can not diffuse through membranes or when endogenous yeast transporters are unable to proceed thus requiring additional transporter overexpression. For instance, overexpressing the malic transporter Mae1 from *Schizosaccharomyces pombe* results in an increase of dicarboxylic acids production in *S. cerevisiae* (Darbani et al., 2019). Finally, playing with organelles size can also be helpful as reported for peroxisome (Kulagina et al., 2021b) and endoplasmic reticulum whose size can be increased through INO2, a regulatory gene in yeast phospholipid biosynthesis, overexpression (Kim et al., 2019) or PAH1 mutation (Arendt et al., 2017), resulting in an optimized triterpene biosynthesis.

## Boosting the cofactor supply

Almost all biosynthetic pathways of plant natural products encompass enzymes needing co-factors such as the cytochromes P450 and alcohol dehydrogenases requiring nicotinamide adenine dinucleotide phosphate (NADPH), *N*- and *O*-methyltransferases requiring *S*-adenosyl-l-methionine (SAM) as well as acyltransferases requiring acetyl-Coenzyme A for instance. All these cofactors thus constitute limiting elements especially when numerous heterologous enzymes are overexpressed in cell factories. So far, strategies aiming at optimizing co-factor supply have already been described such as for producing squalene, an ergosterol pathway intermediate of interest in pharmaceutical and cosmetic industries (Paramasivan and Mutturi, 2017). To compensate the high demand of NADPH of HMGCoA reductase, authors overexpressed regenerating enzyme encoding genes such as ZWF1, a glucose-6-phosphate dehydrogenase and POS5, an NAPH kinase, which resulted in an increase of the bioconversion yields. Similarly, the effect of several NADPH-regenerating enzymes has been tested in yeast for the production of tropane alkaloids (Srinivasan and Smolke, 2020). Similar overexpressions of biosynthetic enzymes have been also carried out to increase SAM amounts inside yeast cells (Zhao et al., 2016).

For the production of podophyllotoxin precursors CaA and FA, Chen and coworkers first recreated a biosynthetic route starting from *p*-coumaric acid (*p*CA) whose synthesis is achieved from deamination of the aromatic amino acid tyrosine and phenylalanine (Li et al., 2015) (Figure 1). Several strategies have been applied to increase *p*CA synthesis up to 130.8 ± 17.0 mg L^−1^. This first includes the improvement of the phenylpropanoid precursors availability through both strengthening the shikimate pathway by overexpressing alternative biosynthetic enzymes and reducing aromatic amino acid consumption *via* knocking-out genes encoding the phenylpyruvate decarboxylase (ARO10) and the pyruvate decarboxylase (PDC5). This was also combined to the direct exploitation of the tyrosine and phenylalanine precursors through overexpression of tyrosine ammonia lyase (TAL) and phenylalanine ammonia lyase (PAL) as well as the heterologous expression of a plant P450s complex containing two cinnamic acid 4-hydroxylases (C4H), a coumarate-3-hydroxylase (C3H) associated with a cytochrome P450 reductase (CPR). *In planta*, C3H mainly catalyzes the hydroxylation of shikimate esters but can also ensure conversion of *p*CA into CaA at low efficiency as reported in yeast. To overcome this limit, authors co-expressed an alternative pCA to CaA direct conversion step from bacteria composed of a FAD (H_2_)-dependent 4-hydroxyphenylacetate 3-monooxygenase (HpaB) and a NADH-dependent flavin oxidoreductase (HpaC), which enabled a CaA production of 157.5 ± 1.2 mg L^−1^. Next, besides overexpressing upstream genes of the pathway such as those encoding shikimate dehydrogenase and chorismate synthase, higher yields of CaA were obtained by increasing the amount of available NADPH fulfilled by the pentose phosphate pathway (Figure 1). Instead of overexpressing NADPH regenerating enzymes which are not always limiting, optimization was achieved through pulling the biosynthetic flux of the pentose phosphate pathway by overexpressing the downstream genes in order to favor NADPH regeneration. This “tour-de-force” was achieved by expressing phosphoketolase (Xfpk) splitting sugars such as fructose 6-phosphate and or xylose 5-phosphate into acetylphosphate and E4P for instance. Furthermore, this last compound has been also efficiently converted to acetyl-CoA by phosphotransacetylase (Pta) with the deletion of GAP phosphatase (Gpp1) to favor growth as previously reported (Liu et al., 2019). A last optimization step was achieved by expressing a native transaldolase (Tald) to further drag flux toward E4P. These combined approaches increased CaA synthesis by 45%, reaching more than 360 mg L^−1^, definitively confirming that optimizing NADPH regeneration *via* pentose phosphate pathway pulling is a key strategy to improve CaA titers.

Besides NADPH, expressing HpaB and HpaC also requires boosting the supply of FAD (H_2_) to improve CaA synthesis. Authors thus deployed three strategies for enhancing FAD (H_2_) availability: 1) consolidating the native FAD (H_2_) synthesis that operates in both the cytosol and mitochondria, 2) managing the export of FAD from mitochondria to the cytosol and 3) implementing the bacterial FAD (H_2_) biosynthetic pathway in yeast cytosol (Figure 1). Firstly, the cytosolic GTP hydrolase II (Rib1) which is the first-rate limiting step of FAD (H_2_) synthesis as well the mitochondrial transporter FLX1 ensuring FA export to the cytosol have been overexpressed resulting in a 51,7% increase in the CaA titer. Then, to alleviate the allosteric inhibition of Rib1 by FAD (H_2_), the bacterial RibBA displaying both guanosine triphosphate (GTP) cyclohydrolase II and 3,4-dihydroxy-2-butanone-4-phosphate synthase activities was implemented in yeast together with the bifunctional riboflavin kinase/FAD synthase (BiFads) that catalyze the conversion of riboflavin into FAD (H_2_) in bacteria. Both expressions resulted in a 54% improvement of the CaA production. When implemented in the strain displaying an enhanced NADPH supply, those strategies were also successful regarding CaA production and were further enhanced by using strong and constitutive promoters for driving gene expression as well as the yeast cultivation in rich medium. The resulting improvement in the accumulation of CaA and its biosynthetic intermediate definitive confirms that the improvement of both the NADPH and FAD (H_2_) supply is of prime importance to boost NP production.

The synthesis of FA relies on the *O*-methylation of CaA which can be catalyzed by different orthodiphenol-*O*-methyltransferases (Omt) including the highly efficient CaA *O*-methyltransferase from *Nicotiana benthamiana* (NtCOMT1) using SAM as a cofactor (Figure 1). While increasing the NtCOMT1 gene copy number together with NADPH and FAD (H_2_) enhanced supply resulted in doubling the FA production, the remaining amount of CaA suggested that the amount of SAM could be limiting in strains highly producing CaA. Interestingly, the classical strategies for optimizing SAM supply such as the expression of rate-limiting methionine adenosyltransferase did not show any huge effect on effect on FA synthesis. By contrast, SAM recycling opened new avenues. Keeping in mind that *S*-adenosyl-l-homocysteine (SAH), which is a SAM by-product resulting from transmethylation, is a potent inhibitor of Omt activity, Chen and coworkers induced the degradation of SAH by overexpression of the SAH hydrolase (Sah1) thus producing homocysteine and adenosine (Figure 1). Since the thermodynamic equilibrium of Sah1 may favor the synthesis of SAH instead of its degradation, the consumption of adenosine has been enhanced through the co-expression of an adenosine kinase (Ado1), thus resulting in a 39% increase in FA production and a 64% conversion of CaA. Finally, such a concrete improvement of the three cofactors supply resulted in a production of 3.8 ± 0.3 g L^−1^ of FA when cultivating yeasts in fed-batch fermentation, thus constituting the best production reported to date.

## Concluding remarks

While many metabolite productions in microbial cell factories did not reach industrial scales so far, the optimization of the biosynthetic fluxes represent one of the key steps in the deployment of the metabolic engineering-based approaches for the sustainable supply of valuables NPs. While many strategies to this aim have been previously developed, Chen and coworkers have demonstrated the prime importance of managing the supply of the cofactors used by the heterologous expressed enzymes. Their integrated strategy based on the synthesis and recycling of three distinct cofactors and ranging from recycling enzyme overexpression, subcellular pathway reorganization as well as inhibitory by-product degradation, resulted in dramatic increase of the CaA and FA synthesis. Besides paving the way for the high production of podophyllotoxin and other lignan derived compounds, this strategy combined to the classical optimization approaches will undoubtedly be used to improve the synthesis of many other NPs requiring co-factors in the near future.

## Perspectives

In line with this recent improvement, yeast cell factories have recently developed for the *de novo* production of the highly valuable alkaloid vinblastine used in several chemotherapy (Zhang et al., 2022). This represents the longest biosynthetic pathway to be transferred from plant to a microorganism. Several additional genetic edits have been also performed to favor alkaloid synthesis including deletion of genes responsible for syphoning biosynthetic intermediates to shunt products. Interestingly, this biosynthetic pathway includes no less than seven different cytochromes P450 and 3 methyltransferases. It is thus temping to speculate that a combined enhancement of both NADPH and SAM supply would greatly increase the production of alkaloids in newly engineered yeast cells. In addition, besides being used as a potential platform for lignan production, the yeast strain developed by the Zhou’s group can be readily valorized for the synthesis of vanillin the most popular flavor compound in the world. Indeed, vanillin synthesis only relies on the metabolization of FA by a hydratase/lyase type enzyme designated vanillin synthase (Gallage et al., 2014). It is therefore obvious that overexpressing this enzyme in the engineered yeast strain would results in high vanillin production amounts. Finally, from a broader point of view, considering non-model organisms for the transfer of biosynthetic pathways could also be of high interest. This is notably the case for *Corynebacterium glutamicum* that can produce high levels of *p*CA (Mhatre et al., 2022).
